# Early Hearing Detection and Intervention programmes for neonates, infants and children in non-Asian low-income and middle-income countries: a systematic review

**DOI:** 10.1136/bmjpo-2024-002794

**Published:** 2024-11-05

**Authors:** Keerthana Rajanbabu, Deepashree Joshi B, Vidya Ramkumar, Hannah Kuper, Ramya Vaidyanath

**Affiliations:** 1 Department of Audiology, Sri Ramachandra Faculty of Audiiology and Speech Language Pathology, Sri Ramachandra Institute of Higher Education and Research (Deemed to be University), Chennai, Tamil Nadu, India; 2 Department of Population Health, London School of Hygiene and Tropical Medicine Faculty of Public Health and Policy, London, UK

**Keywords:** Audiology, Child Health, Deafness, Health services research, Low and Middle Income Countries

## Abstract

**Introduction:**

Early Hearing Detection and Intervention (EHDI) programmes were established to reduce the impact of hearing loss on children. High-income countries (HICs) have resources and knowledge to execute these programmes. However, financial and other resource constraints limit the availability of these programmes to low-income and middle-income countries (LMICs). Yet, LMICs have explored strategies to implement EHDI programmes in their context; the outcomes are still largely unknown.

The aim of this study is to identify the various models of the EHDI program implemented in non-Asian LMICs.

**Aim:**

**Method:**

Studies published between 2010 and 2023 reporting EHDI programmes in non-Asian LMICs for children were considered. The primary databases searched were PubMed, Scopus, Web of Science, EBSCOHost, EBSCO-CINAHL and ProQuest dissertations. The search results are summarised using the Preferred Reporting Items for Systematic Reviews and Meta-Analyses chart. Quality appraisal and risk-of-bias assessment were assessed. Using the retrieved data, a narrative synthesis of the identified methods and forest plots for the prevalence estimate was created.

**Results:**

Fifty-six studies from 16 LMICs were included. They were grouped into 29 hearing screening programmes for neonates and infants and 26 programmes for older children. Predominantly hospital-based screening was employed for neonates and infants and school-based screening for older children. Two-stage otoacoustic emissions screening was employed for neonates and infants, while single-stage pure tone audiometry with otoscopy screening was used for older children. Predominantly, audiologists performed screening and diagnostics for neonates/infants while community health workers performed screening for the older children. Screening aspects were reported predominantly and not diagnostic evaluation/intervention outcomes. Overall, the economics of EHDI was reported only anecdotally in a few studies.

**Conclusion:**

The screening strategies were not uniform among non-Asian LMICs. The protocols used were similar to HICs, yet few developed protocols adapting the Joint Committee of Infant Hearing. However, long-term outcomes such as rate of identification, suitable intervention and their outcomes are not known. EHDI programmes with successful outcomes of early intervention must be studied and reported with economic evaluations.

**WHAT IS ALREADY KNOWN ON THIS TOPIC**

- Early Hearing Detection and Intervention (EHDI) programmes are established as a part of government/public national-level programmes in many high-income countries (HICs) across the world. These programmes in HICs predominantly follow standardised protocol given by Joint Committee of Infant Hearing, which is uniform throughout the country.
- However, a recent publication of systematic review on EHDI programmes of Asian low-income and middle-income countries (LMICs) highlighted that the EHDI programmes are not mandated in many countries. Attempts are made to implement national-level EHDI programmes which are not currently available.

**WHAT THIS STUDY ADDS**

- Non-Asian LMICs have also attempted EHDI programmes but have not been mandated in many countries.
- These programmes largely follow that of HICs, although some LMICs have adapted their own protocols, leading to a lack of uniformity within countries.
- This review has identified the various EHDI programmes implemented in non-Asian LMICs, including the protocol, tools used, screening personnel, site of screening, diagnostic and intervention aspects.
- It also highlights the innovative strategies such as mHealth and tele-health based screening programmes that LMICs have attempted to strengthen their EHDI programmes.

**HOW THIS STUDY MIGHT AFFECT RESEARCH, PRACTICE OR POLICY**

- The review findings can aid the stakeholders and policy-makers in the LMICs to develop or adopt innovative strategies and implement sustainable EHDI programmes with uniform protocols.
- This review also highlights the need for more studies focusing on long-term outcomes of EHDI programmes in LMICs such as diagnostic, intervention and cost outcomes.

## Introduction

 Hearing loss affects an estimated 430 million individuals worldwide,^1^ including 34 million children under the age of 15 years, with a higher prevalence in low-income and middle-income countries (LMICs).^2^ A recent systematic review suggests that the prevalence of hearing loss among children is 1% in LMICs.^3^ However, this is likely an underestimation, as the findings are based on studies with heterogeneous data and a non-representative sample size. Furthermore, 75% of the most common causes of hearing loss in LMICs are reported to be preventable, compared with 46% in high-income countries (HICs).^4^ Among the preventable causes, nearly 60% of them are attributed to poor maternal nutrition and hygiene and late detection and treatment of otitis media.^2^

Hearing loss, at any age, substantially influences the affected individual’s interpersonal relationships, mental health, quality of life and financial independence.^5^ Children with untreated hearing loss have the most difficulty learning to communicate, as verbal language and speech development are directly related to hearing abilities. In the long run, this will affect the child’s schooling, employment and overall quality of life.^6^

Early Hearing Detection and Intervention (EHDI) programmes were implemented in several countries worldwide to improve hearing care services.^7^ In HICs, standard protocols for EHDI targeted towards neonatal screening at birth are implemented, but there is limited information or protocol on how EHDI is conducted in LMICs.^8 9^

Universal Newborn Hearing Screening (UNHS) is known to be particularly economically unviable for environments with limited resources.^7 10^ Other socioeconomic factors, health priorities, lack of awareness about early identification of hearing loss and its benefits, and stigma associated with disability identification also influence the implementation and adoption of EHDI in LMICs.^2 11^ Contextual modifications are required to optimise the benefits of such programmes in LMICs.^9^

Similarly, WHO also emphasises the importance of school hearing screening programmes through which acquired, progressive and late-onset hearing loss can be identified early.^2^ When children miss the initial screenings at place of birth like hospitals and public health centres (PHC) or when screening at birth is not implemented universally in their region, screening during immunisation visits and school screenings are the next possible level of early identification. For example, in LMICs (eg, India and China), the target age groups for EHDI are extended to 6–9 years.^12 13^ Similar to newborn programmes, these programmes are still limited in LMICs.^14^

Despite these challenges, LMICs have also explored hearing screening programmes using strategies within their settings to support their implementation.^14^,^17^ There is a dearth of data on the incidence and prevalence of hearing loss and the specific methods (protocol, screening tools, screening personnel and screening site) of identification and management in these contexts and the outcomes of such efforts.^9 18^ Such data will provide a perspective regarding these countries’ successful and sustainable strategies.

A systematic review was recently published on Asian LMICs.^19^ This parallel systematic review aimed to identify the various models of EHDI programmes for children implemented in non-Asian LMICs. The specific objectives were to identify the various strategies (hospital-based screening, community or school-based screening), screening methods (age of screening, protocol followed (one step/two-step), tools used, personnel involved, use of Information and Communication Technology (ICT) such as e/mHealth tools or databases), diagnostic methods (refer rate and follow-up rate for diagnosis, age of identification, identification rate, tests used, testing site and personnel involved) details of intervention (follow-up rate for intervention, age of intervention, intervention rate and type of intervention) and the cost outcomes of EHDI programmes in these countries.

## Methodology

The protocol for this systematic review was registered in the International

Prospective Register of Systematic Reviews (registration number CRD42021240341).

### Patient and public involvement

No patient involved as it is a systematic review.

### Inclusion criteria

Studies conducted in the non-Asian LMICs that include the regions of sub-Saharan Africa, Middle East and Pacific and Latin America-Caribbean and published between 2010 and 2023 in the English language were considered for this review. All types of quantitative study designs, including cross-sectional, cohort, case-control, randomised control trials and descriptive studies, were included. Studies that involved hearing screening programmes for neonates, infants and children below 6 years of age were considered. The review also included studies that screened children below 6 years of age in their overall representative sample group, which may have extended beyond the age limit. Studies that reported screening protocols, tools, personnel involved and the setting of EHDI programmes in the context of non-Asian LMICs were considered. The review included programmes identifying any type and degree of hearing loss (not restricted to permanent hearing loss).

### Search strategy

PubMed, Scopus, Web of Science, EBSCO Host, EBSCO–CINAHL and Google Scholar were the primary databases searched. The search was conducted from late 2022 till mid-2023 for studies between January 2010 to March 2023. In addition to databases, hand searching was done in the International Journal of Audiology (2010–2023). Grey literature searches included ProQuest Dissertations and the first 500 Google Search results for articles/reports. A pilot search was undertaken in the PubMed and Scopus databases to find the keywords. Keyword synonyms and Medical Subject Headings terms were also identified and incorporated into the search technique. The population/intervention/outcome (PIO) format was used for the search terms (online supplemental table S1).

**Figure 1:**
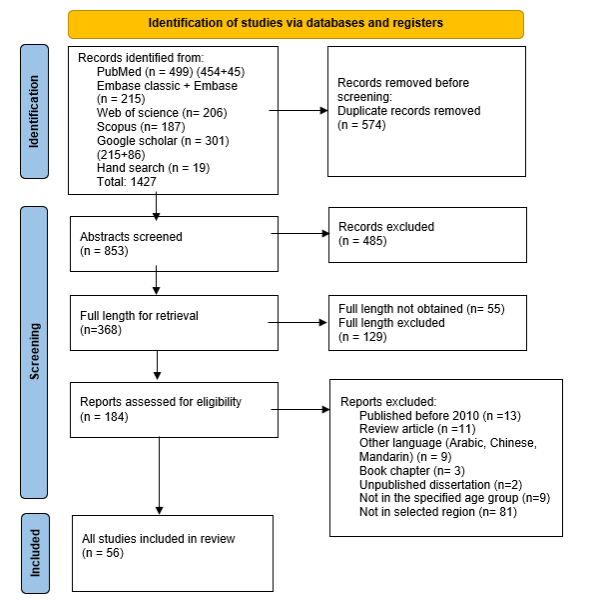
Preferred Reporting Items for Systematic Reviews and Meta-Analyses flow chart representing the selection of article at each stage.

Title screening was carried out as the first step by two reviewers in accordance with the inclusion criteria for each database. The search results were extracted into Rayyan software^20^ for duplicate removal. Once the duplicates were removed, the next stage had two reviewers screened the abstracts and full texts using the same software. When full-length papers were not available, an email was sent to the corresponding authors. These articles were removed if no response was received. Any disagreements were resolved by discussion among the reviewers. The search results were represented using the Preferred Reporting Items for Systematic Reviews and Meta-Analyses (PRISMA) flow chart.^21^ Figure 1 depicts the screening phases and the number of articles chosen at each level.

### Data extraction and synthesis

The data related to age of screening, screening method, screening procedure, tools used, usage of ICT (e/mHealth tools or databases), person performing screening, refer rate (number of children referred from screening stage to diagnostic testing), diagnostic site and personnel, diagnostic tool, identification rate (number of children identified to have hearing loss out of the total screened population), intervention rate (number of children who received intervention out of the children identified with hearing loss) and economic analysis of the programme were all retrieved. The programmes’ reported limitations were also documented. The data were extracted from Google Sheets, and a narrative synthesis of the frequency distribution was performed based on the objectives. The regionwise prevalence (per 1000) of hearing loss was estimated using a random effect forest plot for regions with three or more studies reporting hearing loss data.

### Quality and risk-of-bias assessment

The Critical Appraisal Skills Program (CASP)^22^ checklist was used to evaluate the quality of the studies included based on research design. Risk-of-bias assessment (RoB)^23^ was conducted by two reviewers using the results of CASP. The questions answered with ‘yes’ were considered ‘low bias’, ‘don’t know’ was considered ‘unclear’ and ‘no’ was considered ‘high bias’. The overall bias was considered as ‘low’ if no/one item was rated as ‘unclear/high bias’. If there were two items with ‘unclear/high bias’, then the overall bias was considered as ‘medium’ and if there were three or more items with ‘unclear/high bias’, then the overall bias was considered as ‘high’. This criterion for labelling overall bias as high/medium/low was given by the reviewers.

## Results

A total of 1427 studies were identified during the electronic search. Following the abstract and full-length screening, 56 studies qualified for the review. The PRISMA flow chart depicts the selection procedure (figure 1). The studies were classified based on the age of screening, such as *hearing screening programmes for neonates and infants* for 0–1 year or *hearing screening programmes for older children* for >1–6 years. The summary of data extracted from hearing screening programmes for neonates and infants is provided in table 1 and for older children in table 2.

**Table 1:** Hearing screening programmes for neonates and infants in non-Asian low-income and middle-income countries

Citation	First author and year	Country	No of samples screened	Service delivery model (hospital/community/school)	Screening test/tool used (stage 1+ stage 2+ stage 3)	Person performing screening	Person performing diagnostic evaluation	Diagnostic test used	Site of diagnostic testing	Intervention details (medical/surgical/rehab)	Refer rate	Identification rate
**Sub-Saharan Africa**												
76	Ndoleriire *et al*, 2023	Uganda – Sub–Saharan Africa (LI)	1217	Hospital	TEOAE+TEOAE	Midwives	Audiology technicians	Diagnostic ABR+OAE + Tymp	Hospital	NA	NA	3.6% (2.2% unilateral, 1.4% bilateral)
32	Werkineh *et al*, 2022	Ethiopia – Sub–Saharan Africa (LI)	368	Hospital	TEOAE+TEOAE If refer, TEOAE+AABR	Audiologist	NA	Diagnostic ABR+OAE + Tymp	Hospital	NA	15.5%	NA
45	Abdullahi *et al*, 2022	Nigeria – Sub–Saharan Africa (LMI)	150	Hospital	Otoscopy/TEOAE	Audiologist	NA	NA	NA	NA	8%	NA
36	Seguya *et al*, 2021	Uganda – Sub–Saharan Africa (LI)	401	Hospital	TEOAE+TEOAE+AABR	Nurse, ENT Resident	NA	NA	NA	NA	25.4%	1.10%
54	Bezuidenhout *et al*, 2021	South Africa – Sub–Saharan Africa (UMI)	121	Hospital	(DPOAE) + (Otoscopy/DPOAE/tymp)	Audiologist	Audiologist	Diagnostic ABR	Tertiary academic hospital	NA	47%	NA
33	Gina *et al*, 2021	South Africa – Sub–Saharan Africa (UMI)	2269	Hospital	OAE/ABR	Audiologist	Audiologist	NA	NA	NA	ABR: 6.17%OAE: 71%	NA
46	Er *et al*, 2020	Ghana – Sub–Saharan Africa (LMI)	483	Hospital	DPOAE	Audiologist	NA	NA	NA	NA	5.5%	7.20%
34	Kanji *et al*, 2018	South Africa – Sub–Saharan Africa (UMI)	272	Hospital (PHC, MOU, nursery)	Nursery and MOU: (Otoscopy/DPOAE) PHC: (DPOAE/TEOAE) + AABR	Audiologist	NA	NA	NA	NA	MOU:0.74%PHC:36%	NA
24	Bezuidenhout *et al*, 2018	South Africa – Sub–Saharan Africa (UMI)	121	Hospital	(Otoscopy/DPOAE) + (otoscopy/DPOAE/AABR/tymp)	Audiologist	Audiologist	NA	Audiology clinic	NA	47.1%	NA
37	Kock *et al*, 2016	South Africa – Sub–Saharan Africa (UMI)	7452	Community (MOU)	DPOAE+AABR	Trained non specialist screener	Audiologist	NA	Hospital	NA	AABR: 4.6%DPOAE: 7.0%	NA
25	Kanji *et al*, 2016	South Africa – Sub–Saharan Africa (UMI)	11	Hospital	OAE+AABR	NA	Audiologist	Behavioural assessment (VRA)	NA	NA	45.45%	NA
47	Walsh *et al*, 2015	Uganda – Sub–Saharan Africa (LI)	60	Hospital (Local health centres)	TEOAE	NA	NA	Diagnostic ABR+OAE	Paediatric ear clinic	NA	10%	NA
48	Dyk *et al*, 2015	South Africa – Sub–Saharan Africa (UMI)	150	Hospital	TEOAE+TEOAE + AABR	Audiologist	NA	NA	NA	NA	AABR: 16.7%TEOAE: 37.9%	NA
35	Khoza-Shangase *et al*, 2015	South Africa – Sub–Saharan Africa (UMI)	272	Community (MOU)	(Otoscopy/DPOAE) + (Otoscopy/DPOAE)	Audiologist	NA	NA	NA	NA	11.14%	NA
53	Lasisi *et al*, 2014	Nigeria – Sub–Saharan Africa (LMI)	453	Hospital	AABR+AABR	Audiologist	NA	NA	NA	NA	44.7%	NA
75	Friderichs *et al*, 2012	South Africa – Sub–Saharan Africa (UMI)	NA	Hospital (Immunisation, PHC)	DPOAE+DPOAE	Non specialist, nurses	Audiologist	NA	Hospital	NA	9.5%	4.5% (3% CHL, 1.5% SNHL)
78	Tanon-Anoh *et al*, 2010	Cote d’Ivoire – Sub–Saharan Africa (LMI)	1306	Hospital	TEOAE+TEOAE	Trained specialist	Audiologist	Diagnostic ABR+OAE + Tymp	Hospital	NA	21.9%	5.96%
**Latin America-Caribbean**												
50	Chiriboga *et al*, 2021	Brazil – Latin America and Caribbean (UMI)	9941	Hospital	Well babies: TEOAE+TEOAE + TEOAE NICU: (TEOAE/AABR)	Audiologist	Audiologist	NA	Hospital	NA	Well babies: 3.12%NICU babies:4.36%	Well babies: 0.04% NICU babies: 1.88%
77	Dutra *et al*, 2020	Brazil – Latin America and Caribbean (UMI)	1380	Hospital	TEOAE+TEOAE	Trained specialist	Audiologist	NA	Hospital	NA	1.9%	NA
51	Marinho *et al*, 2020	Brazil – Latin America and Caribbean (UMI)	3981	Hospital	Well babies: OAE+OAE + OAE HRR: OAE+ABR	NA	NA	NA	NA	NA	4.2%	NA
38	Olarte *et al*, 2019	Colombia – Latin America (UMI)	1646	Hospital	TEOAE+TEOAE	Audiologist	Audiologist	NA	Hospital	NA	1.6%	NA
49	Ospina-Garcia *et al*, 2019	Colombia – Latin America (UMI)	962	Hospital	OAE+OAE + OAE	Audiologist	Audiologist	Diagnostic ABR	Hospital	NA	7.17%	4.4% (0.42% SNHL, 3.1% unilateral, 1.1% bilateral)
56	Wong *et al*, 2017	Nicaragua – Latin America (LMI)	640	Hospital	(OAE) + (OAE/tymp)	Audiologist	Audiologist	Diagnostic ABR	Hospital	NA	5.94%	NA
57	Lima *et al*, 2015	Brazil – Latin America and Carribean (UMI)	757	Hospital (maternity hospital)	(DPOAE/cochlear eyelid reflex) + (DPOAE and Cochlear eyelid reflex)	Nurses	Audiologist	Diagnostic ABR+Tymp	NA	NA	1.8%	NA
26	Bevilacqua *et al*, 2010	Brazil – Latin America and Caribbean (UMI)	11 466	Hospital	TEOAE+TEOAE	Audiologist	NA	Diagnostic ABR+ASSR	Speech and hearing clinic	HA fitting: 8 children; rehabilitation process: 5 children	22.3%	0.096% (0.01% CHL, 0.002% SNHL)
**Middle East and North Africa**												
55	Omar *et al*, 2022	Egypt – Middle east and North Africa (LMI)	200	Hospital	(Otoscopy/TEOAE) + TEOAE	NA	NA	Diagnostic ABR	Tertiary care hospital	NA	4.5%	1%
79	Nuseir *et al*, 2021	Jordan – Middle east and North Africa (UMI)	1595	Hospital	(TEOAE/DPOAE) + (TEOAE/DPOAE)	NA	NA	Diagnostic ABR	Hospital	NA	6.04%	0.12%
52	Saki *et al*, 2017	Iran – Middle East and North Africa (UMI)	92 521	Hospital	TEOAE+AABR	Audiologist	Audiologists	Diagnostic ABR+OAE	Hospital	NA	1.25%	0.24% (5% CHL, 87% SNHL)
27	Imam *et al*, 2013	Egypt – Middle east and North Africa (LMI)	150 (100 NICU babies and 50 full-term)	Hospital	TEOAE+TEOAE	Audiologist	Audiologist	Diagnostic ABR+Tymp	Hospital	Medical management (resolution of middle ear effusion)	NICU: 34%Well babies: 16%	NA

AABRautomated auditory brainstem responseABRauditory brainstem responseASSRauditory steady-state responseCHLconductive hearing lossDPOAEdistortion product otoacoustic emissionsHAhearing aidsLIlow incomeLMIlower middle incomeMOUmidwife obstetric unitsNAnot availableNICUneonatal intensive care unitsOAEotoacoustic emissionsPHCpublic health centerSNHLsensorineural hearing lossTEOAEtransient evoked otoacoustic emissionsTymptympanometryUMIupper middle incomeVRAvisual reinforcement audiometry

**Table 2:** Hearing screening programmes for older children in non-Asian low-income and middle-income countries

Citation	First author and year	Country	Age in years	No of samples screened	Service delivery model (hospital/community/school)	Use of ICT in any phase of model	Screening test/tool used (stage 1+ stage 2+ stage 3)	Person performing screening	Person performing diagnostic evaluation	Diagnostic test used	Site of diagnostic testing	Intervention details (medical/surgical/rehab)	Refer rate	Identification rate	Rehab rate
**Sub-Saharan Africa**															
69	Eksteen *et al*, 2022	South Africa - Sub-Saharan Africa (UMI)	4–7 years	10 390	Community	mHealth app-based screening Cloud-based data storage management	PTA screening (HearScreen)	CHWs	Audiologist	Otoscopy, PTA	School	HA, medical management, Healthcare centre	5.6%	2.30%	NA
28	Shinn *et al*, 2021	Kenya - Sub-Saharan Africa (LMI)	2–16 years	127	School	No	PTA screening (HearScreen)	Teachers	NA	NA	NA	NA	20%	6.20%	NA
70	Dawood *et al*, 2020	South Africa - Sub-Saharan Africa (UMI)	3–10 years	6805	School	mHealth app-based screening Cloud storage data management	PTA screening (HearScreen)	Nurses, CHWs	NA	NA	NA	NA	3–5 years: 7.6% 6–10 years: 4%	NA	NA
65	Larsen-Reindorf *et al*, 2019	Ghana - Sub-Saharan Africa (LMI)	3–15 years children	341	Community	mHealth based screening	LittleEars auditory questionnaire/otoscopy/PTA screening (Shoebox tablet audiometer)	Audiology students, audiologist	Audiologist	NA	Hospital	Medical management (wax removal)	2.2%	1.1% (0.4% CHL, 0.7% SNHL)	NA
39	Shinn *et al*, 2019	Kenya - Sub-Saharan Africa (LMI)	2–16 years children	104	School	mHealth app-based screening	PTA screening (HearX)	CHWs	Audiologist	NA	NA	NA	7%	NA	NA
66	Yancey *et al*, 2019	Kenya - Sub-Saharan Africa (LMI)	5–16 years (suspected with HI)	155	School	Cloud storage data management (Electronic medical record)	Video otoscopy/PTA screening	Nurses, CHWs	CHWs monitored by ENT doctors	PTA	NA	Medical and surgical management, Hospital	18%	10%	NA
58	Eksteen *et al*, 2019	South Africa - Sub-Saharan Africa (UMI)	4–7 years	8023	School	mHealth app-based screening Cloud storage data management	PTA screening (HearScreen)+ Otoscopy	CHWs, Audiologist	Audiologist	PTA	NA	HA, spectacles, medical management, Public health services	5.4%	0.70%	NA
43	Osei *et al*, 2018	Ghana - Sub-Saharan Africa (LMI)	5–17 years	210	School	No	Otoscopy/PTA screening	Audiologist, Audiology technician	NA	NA	NA	Medical management (wax removal, middle ear pathology management)	21%	11.90%	NA
31	Jayawardena *et al*, 2018	Kenya - Sub-Saharan Africa (LMI)	Any age group	174 ears from 87 patients	Community screening (head and neck surgical mission)	No	Otoscopy/PTA screening	Nurses, CHWs	Nurses, CHWs	PTA	NA	NA	NA	61.43%	NA
29	Govender *et al*, 2018	South Africa - Sub-Saharan Africa (UMI)	6–12 years	146	School screening	No	Otoscopy/PTA screening (Kuduwave automated audiometry) + tymp	Trained non-specialist	Audiologist	PTA	NA	NA	NA	16% (12% unilateral, 4% bilateral)	NA
59	Yousuf Hussein *et al*, 2018	South Africa - Sub-Saharan Africa (UMI)	3–6 years	6424	Community	Asynchronous screening Cloud-based data storage management	PTA screening+PTA screening (HearScreen)	CHWs	NA	NA	Local clinic	NA	40.5%	NA	NA
71	Yousuf Hussein *et al*, 2018	South Africa - Sub-Saharan Africa (UMI)	Preschool children	6424	Community (early childhood development centres)	mHealth app-based screening Cloud-based data management	PTA screening (HearScreen)	CHWs	Audiologist	NA	Local clinic	Medical management (wax removal)	24.9%	18.7% (65.2% CHL, 28.2% SNHL, 6.5% MHL)	9.3% were treated for cerumen
60	Tataryn *et al*, 2017	Malawi - Sub-Saharan Africa (LI)	School children	7220	Community	Yes (no information)	Less than 5 - questionnaire+OAE More than 5 - PTA screening+ otoscopy	ENT clinical officer, Audiologist	NA	NA	NA	NA	NA	27%	NA
67	Hunt *et al*, 2017	Malawi - Sub-Saharan Africa (LI)	4–6 years	281	Community	mHealth app-based screening	Questionnaire/video otoscopy/PTA screening (HearScreen)	ENT clinical officer	Trained non-specialist monitored by ENT doctors	NA	Tertiary hospital	Medical management (wax removal)	NA	46% (24.5% unilateral, 12.5% bilateral)	NA
72	Mahomed Asmail *et al*, 2016	South Africa - Sub-Saharan Africa (UMI)	Grade 1–3 children	1070	School	No	(Otoscopy/PTA screening/tymp)	Audiology students	Audiologist	PTA	NA	NA	11.6%	2.20 (57.1% CHL, 22% MHL, 20% SNHL, 37.1% unilateral, 31.4% bilateral)	NA
40	Simões *et al*, 2016	Kenya - Sub-Saharan Africa (LMI)	2–15 years	13 109	School	No	(Otoscopy/tymp) + PTA screening	ENT clinical officer	ENT clinical officer	PTA	School	Surgical management (perforated ear drums), HA	NA	1.50%	NA
41	Adedeji *et al*, 2015	Nigeria - Sub-Saharan Africa (LMI)	1–15 years age children with HL	223	Hospital	No	PTA screening/AABR	NA	NA	PTA, ABR	Hospital	CI for profound HI, HA fitting with auditory and speech training, 20.6% had enrolled at school for the deaf	67.%	NA	Less than 5% had HA followed by auditory and speech training
73	Govender *et al*, 2015	South Africa - Sub-Saharan Africa (UMI)	Grad1 toddlers	378	School	No	(Otoscopy/PTA screening/tymp)	Audiology students	Audiologist	NA	Hospital	Medical management (wax removal, middle ear pathology management)	NA	NA	NA
30	Cloete *et al*, 2015	South Africa - Sub-Saharan Africa (UMI)	6–7 years	100	School	No	DPOAE screening	Nurses	Audiologist	PTA	Hospital	NA		NA	NA
68	Adebola *et al*, 2013	Nigeria - Sub-Saharan Africa (LMI)	3.5–6 years	101	School	No	Otoscopy/PTA screening	ENT doctor, Audiologist	NA	NA	NA	Medical management (wax removal, middle ear pathology management)	21.3%	NA	Improved to 88.6% otoscopic pass rate after ENT intervention
**Latin America and Caribbean**															
61	Urban *et al*, 2022	Dominican Republic - Latin America and Caribbean (UMI)	5–7 years	528	Hospital	No	(Otoscopy/PTA screening) + (DPOAE/tymp)	Audiology students	NA	NA	NA	Medical management (myringotomy, pressure equalising tubes), HA candidacy assessment, Hospital	3.8%	1.90%	NA
44	Jayawardena *et al*, 2020	Haiti - Latin America (LI)	5–18 years	127	School screening	No	PTA screening	CHWs	NA	PTA, otoscopy	NA	NA	25%	NA	NA
62	Magro *et al*, 2019	Nicaragua - Latin America (LMI)	School children	120	School	mHealth based screening	PTA screening+PTA screening (WAHTS - automated/manual)	Audiometric technician	Audiometric technician	NA	Hospital	NA	NA	5%	NA
63	Samelli *et al*, 2012	Brazil - Latin America and Caribbean (UMI)	3–6 years	507	School	No	Questionnaire+tymp	NA	NA	NA	NA	NA	38.7%	NA	NA
42	Samelli *et al*, 2011	Brazil - Latin America and Caribbean (UMI)	2–10 years	214	Community	No	Questionnaire/otoscopy/PTA screening/tymp	NA	NA	NA	NA	NA	NA	46% (39.2% CHL, 7.48% SNHL)	NA
**Middle East and North Africa**															
74	Elbeltagy *et al*, 2020	Egypt - Middle east and North Africa (LMI)	6–9 years)	100	School	No	PTA screening/tymp/SIFTER questionnaire	NA	NA	PTA	Hospital	NA	NA	23% (17% CHL, 6% SNHL, 7% Unilateral, 6% bilateral)	NA
64	Mahmoud *et al*, 2016	Egypt - Middle east and North Africa (LMI)	4–7 years	4500	School	No	Otoscopy/PTA screening/tymp) + (otoscopy/PTA screening/tymp)	Audiologist	Audiologist	PTA, ABR	Hospital	NA	20%	5.80%	NA

AABRautomated auditory brainstem responseABRauditory brainstem responsesCHLconductive hearing lossCHWcommunity health workersCIcochlear implantationDPOAEDistortion Product Otoacoustic EmissionsHAhearing aidsHIhearing impairmentLIlow incomeLMIlower middle incomeMHLmixed hearing lossNAnot availablePTApure tone audiometrySNHLsensorineural hearing lossTymptympanometryUMIupper middle income

Studies were obtained from 16 LMICs of the non-Asian regions. The maximum number of studies were reported from sub-Saharan Africa (n=37), followed by Latin America-Caribbean (n=13), and the Middle East and North Africa (n=6). Among the countries in these regions, the maximum number of studies were reported from South Africa (n=19), followed by Brazil (n=7), Kenya (n=5), Egypt and Nigeria (n=4 each), Ghana and Uganda (n=3 each), Columbia, Malawi and Nicaragua (n=2 each), and Cote d'Ivoire, Dominican Republic, Ethiopia, Haiti, Iran and Jordan (n=1 each). More than half (n=31) of the studies were from upper-middle-income countries like Brazil, Columbia, Dominican Republic, Iran, Jordan and South Africa (six countries), followed by lower-middle-income countries (n=18) that include Cote d’Ivoire, Egypt, Ghana, Kenya, Nicaragua and Nigeria (six countries) and the lowest proportion (n=7) were from low-income countries like Ethiopia, Haiti, Malawi and Uganda (four countries).

There were 49 cross-sectional studies, six cohort studies,^24^,^29^ and one study^30^ with mixed methods design. For all cross-sectional studies, the CASP diagnostic study checklist was used after excluding four items (out of 12) on the checklist that were deemed ‘not applicable’. The cross-sectional studies do not have any reference standards or cost alternatives. So, these questions were deemed not applicable. For the cohort studies, the CASP cohort study checklist was used after excluding three items (out of 12) that were ‘not applicable.’

The RoB was assessed using the results obtained from the quality appraisal (online supplemental figure S1). Only one study^25^ had ‘medium bias’ due to unclear data regarding participant characteristics and test descriptions. All the other studies identified in the review had ‘low bias’.

### Hearing screening strategies

There were 29 studies that reported outcomes of hearing screening programmes for neonates and infants, 26 studies on screening programmes for older children and one study that included all ages between 1 years to 64 years.^31^ Neonatal and infant screening occurred in hospitals (n=27), predominantly in PHC, midwife clinics or immunisation clinics, followed by community settings (n=2). Older children were screened at school (n=17), community setting (n=8) or in a hospital setting (n=2).

### Hearing screening methods

#### Age of screening

The hearing screening programmes for neonates and infants were conducted as early as 6 hours after birth,^32^,^35^ to as late as 1 month^2436^,^38^ of age. Hearing screening among older (preschool/school-aged) children was reported from 1 years onwards^2831 39^,^42^ to 17 years.^43 44^

#### Screening protocol and tests

The hearing screening for neonates and infants was mostly two stage (n=19) followed by one-stage screening protocol^3334 45^,^47^ and sometimes even a three-stage protocol.^36 48 49^ Two studies used either a two-stage or one-stage protocol for high-risk babies, while well babies had three screening stages.^50 51^

Otoacoustic emissions (OAE) screening alone (n=17) was the screening tool predominantly used to screen neonates and infants. The use of a combination of OAE and/or automated auditory brainstem response (AABR) was comparatively lesser,^2425 32^,^34 36 37 48 50^ and occasionally AABR alone^53^ was also used. Otoscopy and tympanometry were additionally used in some studies for neonates and infants. While otoscopy was used predominantly in the first stage of screening,^24 34 35 45 54 55^ tympanometry was used during the second stage.^24 54 56^ Additionally, behavioural responses (eyelid reflex) were also observed in one study.^57^

Screening for older children predominantly involved a single-stage screening protocol, while few used a two-stage protocol.^2940 42 58^,^64^ Only one study used a three-stage screening for infants and older children.^44^

Otoscopy and pure tone audiometry were the most used tests for older children,^3143 44 58 60 65^,^68^ closely followed by subjective screening tests alone.^2839 41 59 62 69^,^71^ Pure tone audiometry was predominantly carried out using smartphone-based applications such as HearScreen,^28 58 67 70 71^ HearX applications^39 44^ and KUDUWAVE automated audiometry.^29^ Few studies have reported a test battery, even at the screening stage involving tympanometry, pure tone audiometry and otoscopy.^29 40 42 61 64 72 73^

In addition, validated questionnaires,^42 60 63 67^ SIFTER^74^ and LittleEars questionnaire^65^ were also used for screening for older children. A combination of subjective screening along with OAE,^61^ AABR^41^ and immittance^74^ was used in a few studies, while OAE alone^30^ and immittance^63^ alone were also used occasionally.

#### Screening personnel

Hearing screening for neonates and infants was mostly conducted by audiologists (n=21), followed by nurses,^36 57 75^ and other medically qualified personnel, including otolaryngologists.^33 37 47 76 77^

Screening for older children was frequently conducted by community health workers,^3139 44 58 59 66 69^,^71^ followed by audiologists,^43 58 60 64 65 68^ nurses,^30 31 66 70^ trained volunteers,^29 40 67 68^ internship students^61 65 72 73^ and audiometric technicians.^43 62^ One study also employed a school teacher for screening.^28^ Eight studies did not mention the screening personnel.^41 42 63 74^

A few studies briefly explained the training provided to nurses and other individuals. The training programmes were for 5 days,^58^ half-day,^29 60 76^ or only a few hours^37 66^ and included hands-on training and workshops on how to position the probe, operate the equipment, use the mobile-based application and transfer and store data.

#### Use of ICT

In hearing screening programmes for neonates and infants, only one used the asynchronous e/mHealth model to share data captured post-OAE screening.^57^ In studies that involved older children, ICT was used predominantly for electronic medical records^5859 69^,^71^ to track follow-up. Only a couple of studies used e/mHealth tools for asynchronous screening.^59 67^

### Diagnostic methods

#### Refer rate

The number of children referred from screening among neonates and infants (who failed the screening stage) and monitored for diagnostic follow-up ranged from around 1%–2%^34 38 52 57 77^ to around 40%–50%^24 25 27 48 53 54^ in some studies. One study did not mention this result but directly reported the prevalence.^76^

In older children, the highest was 67%,^41^ followed by 40.5%.^59^ The others varied greatly between 2%^65^ and 38%.^63^ Many directly reported only the diagnostic details.

#### Follow-up rate for diagnostics and age of identification

The follow-up rate for diagnostic testing in hearing screening programmes for neonates and infants ranged from 80%^37 78^ to 100%.^50 52 79^ Among programmes for older children, the follow-up rate varied from 25%–45%^59 65 71^ to 100%.^61^

The age of children identified through the hearing screening for neonates and infants was seldom reported. The age reported was 22 weeks^78^ in one study and 1–13 months^26^ in another study. The age of identification was not mentioned for any older children screening programmes included in this review.

#### Diagnostic tests and testing sites

Fourteen studies on screening programmes for neonates and infants provided some information on the diagnostic tests used. In most programmes, diagnostic ABR alone was used to confirm the presence of hearing loss.^4149 54^,^56 64 79^ Only a few studies used a combination of tests that included tympanometry, ABR and OAE,^32 76 78^ ABR and OAE,^47 52^ or ABR and Auditory Steady State Response (ASSR),^26^ or ABR and tympanometry.^27^ Only 16 of the 29 studies for neonates and infants reported that the diagnostic testing was conducted in a tertiary care hospital (n=16), different from the screening site. This was followed by paediatric hearing clinics or local clinics.^24 26 47^

Of the studies on screening programmes for older children, 12 reported the diagnostic test details. Subjective tests were commonly used for threshold estimation.^2529^,^31 40 41 58 64 66 72^ A combination of tests was performed in some, including pure tone audiometry with tympanometry^57^ or pure tone audiometry with otoscopy.^69^ Few other studies reported that diagnostic testing was carried out but did not specify the tests performed.^59 62 65 67 71 73^ Only eight of 27 studies reported that diagnostic testing was conducted in tertiary care hospitals,^30 41 62 64 65 67 73 74^ followed by paediatric clinics^59 71^ and schools.^40 69^ The remaining 15 studies had no information related to the diagnostic testing.

#### Diagnostic testing personnel

Among the hearing screening programmes for neonates and infants, only 16 studies mentioned the personnel involved in diagnostic testing. Audiologists predominantly conducted diagnostic testing (n=15 studies), and in one study an audiology technician was reported to be involved in diagnosis.^76^

Among studies on hearing screening for older children, 15 mentioned the personnel involved in diagnostic testing. Predominantly, the audiologists^2930 39 58 64 65 69 71^,^73^ performed diagnostic testing. However, community health workers or trained technicians,^31 62 66 67^ nurses^31^ and even otolaryngologists^40^ were reported to conduct diagnostic testing. In some programmes, otolaryngologists monitored the proceedings and diagnosed the data obtained by community health workers or nurses.^66 67^

#### Prevalence of hearing loss

The prevalence data are based on the diagnostic results reported in the studies. The screening programmes for neonates and infants did not describe the degree of hearing loss among those identified. Only four studies from them^26 49 52 75^ specified the type of hearing loss, and two mentioned the proportion of unilateral and bilateral loss.^49 76^ Conductive hearing loss was comparatively higher in prevalence.

**Figure 2:**
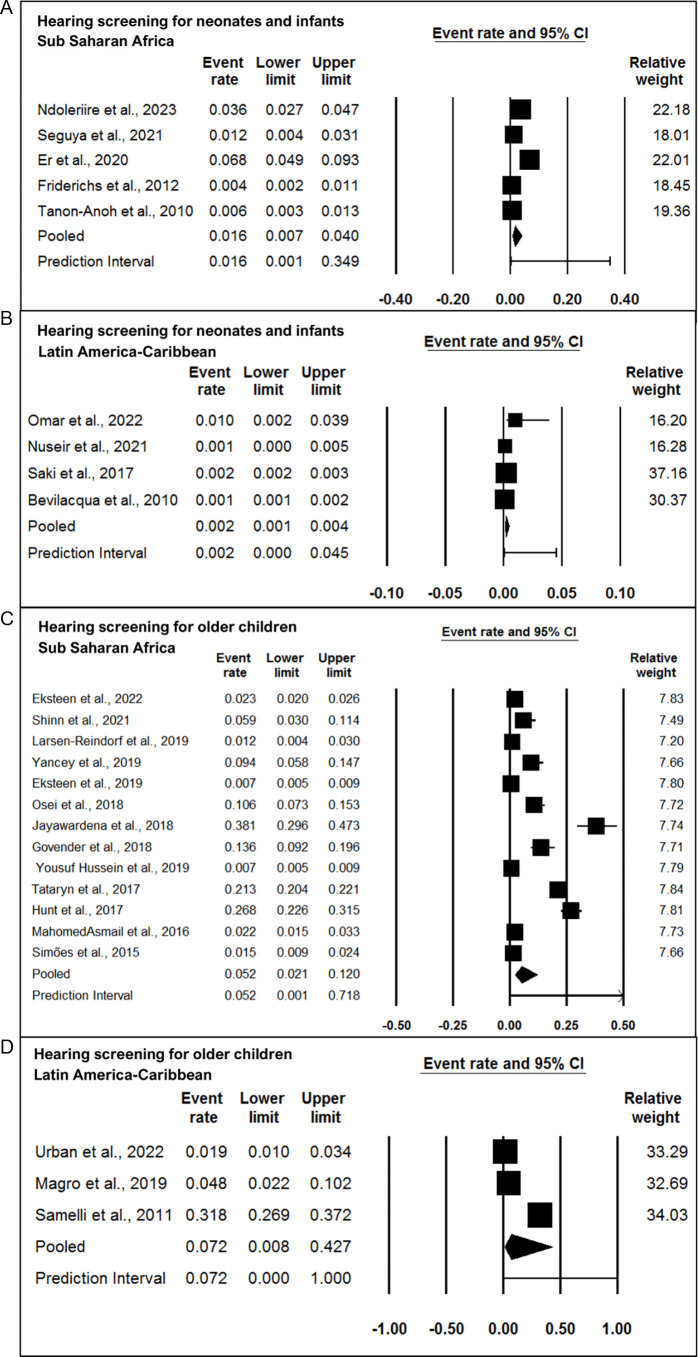
Forest plot of prevalence of hearing loss in (A) hearing screening programmes for neonates and infants sub-Saharan Africa; (B) hearing screening programmes for neonates and infants Latin America-Caribbean; (C) hearing screening programmes for older children sub-Saharan Africa; (D) hearing screening programmes for older children Latin America-Caribbean.

Among the hearing screening programmes for neonates and infants, only 11 studies reported the prevalence of hearing loss, of which five were from the sub-Saharan region^36 46 75 76 78^ and four were from Latin America-Caribbean.^26 52 55 79^ From the forest plot of figure 2a and b (figure 2), the prevalence of hearing loss in the sub-Saharan region was found to be 16 per 1000, and in Latin America-Caribbean was 2 per 1000. The estimated prevalence from the two studies was 4 per 1000 and 2 per 1000 in the Middle East and North Africa.^49 50^ Due to limited studies from the Middle East and North Africa, forest plots could not be prepared.

Among the screening programmes for older children, 16 studies reported the prevalence of hearing loss, of which 13 were from the sub-Saharan region,^2829 31 40 43 58^,^60 65^ three from Latin America-Caribbean^42 61 62^ and two from the Middle East and North Africa.^64 74^ From the forest plot in figure 2c and d (figure 2), the prevalence of hearing loss in the sub-Saharan, Latin American-Caribbean regions is found to be 5 per 1000 and 7 per 1000. As reported by the two studies from the Middle East and North Africa, the prevalence is 6 per 1000 and 23 per 1000, respectively.

Four studies from the older children screening programmes mentioned that mild or mild-to-moderate degrees of hearing loss were the most prevalent.^29 31 42 72^ The exact decibel considered for mild or moderate was not specified in either of these studies. These studies with mild and moderate degrees of hearing loss and that identified conductive hearing losses were also included for forest plot generation. Only five studies from the screening programme for older children specified the type of hearing loss with conductive hearing loss being the most prevalent.^42 65 71 72 74^ The proportion of unilateral and bilateral loss was given in four studies.^29 67 72 74^

### Intervention methods

#### Follow-up rate for intervention and age of intervention

The follow-up rate for intervention was not reported in any hearing screening programmes for neonates and infants. Only one study from the neonates and infants reported the age of intervention to be less than 1 year of age.^26^

Programmes for older children had a follow-up rate of 2%^61^–32%.^73^ However, none of the studies from the programmes reported details of the age of intervention.

#### Types of intervention

Only two hearing screening programmes for neonates and infants reported the recommendations for interventions such as hearing aid (HA), cochlear implant (CI), speech and language therapy,^26^ or medical management for middle ear effusion.^27^ However, the studies did not mention how many followed up or obtained the recommended intervention.

Among the screening programmes for older children, medical interventions recommended were wax removal,^68 71^ myringotomy for tympanic membrane perforation^61^ and provision of HAs.^41^ These interventions were provided within the scope of the programme. Some studies mentioned that they recommended medical intervention,^4058 65^,^67 69 73^ provision of HA or CI based on the candidacy,^26 40 41 58 61 69^ and spoken language therapy to children identified with hearing loss,^26 41^ but follow-up/uptake of these interventions was not reported.

### Economic analysis of the screening programs

Among the hearing screening programmes for neonates and infants, only one study reported that UNHS with OAE and AABR and targeted newborn hearing screening with AABR were cost-effective when certain baseline parameters were optimised.^56^ None of the other studies conducted an economic analysis to provide insights into the cost-outcome, cost-effectiveness or cost-utility of EHDI programmes implemented in these contexts. Studies have anecdotally reported that OAEs were perceived to be more cost-effective than ABR for newborns because it is easier to perform the test.^55 76^ ABR was considered unviable due to recurrent consumable costs.^38 54^ Screening using a tablet or smartphone was considered a low-cost alternative for resource-constrained settings, as reported in a few programmes for older children.^59 62 70^ Employing community workers, clinically trained volunteers and school teachers for screening was another cost-effective method reported.^39 44 59 65 75^

## Discussion

According to the World Bank classification (2021), there are 84 LMICs in sub-Saharan Africa, the Middle East and Latin America-Caribbean, with publications from 16 countries included in this systematic review. The majority of the studies identified in this review were from upper-middle-income countries, which is comparable to the trend seen in Asian LMICs.^19 80^ Therefore, the implementation of EHDI appears to be proportional to the country’s economy. While screening is being implemented, the relevant outcome of attaining early intervention is not yet known from these studies.^14 17 81^ Despite the extension of the age limit to 6 years for LMICs, many studies still included a larger age range for children to have better coverage. Therefore, this review also included older children beyond 6 years.

### Hearing screening strategy

Hospital screening at birth, which included immunisation clinics, maternity units and well-baby care clinics, was the most commonly employed neonatal/infant hearing screening strategy.^11 37 61 76 81^ The contrast in the context of EHDI implementation in HICs must be considered before duplicating hearing screening programmes in LMICs to maintain sustainability and improve early intervention outcomes.^9 10^ For example, there is a considerable shortage of trained individuals in maternity and neonatal health, particularly in rural areas of LMICs^7 8^; births are often performed in remote primary clinics or home settings.^82 83^ To address this gap, the WHO developed criteria for screening tools and protocols customised to the country’s national, cultural and socioeconomic situations in 2021.^10^ In such contexts, community-based initiatives or a combination of hospital and community-based programmes are often recommended.^84^,^87^

Considering the burden of unidentified hearing loss, school-based screening has been increasingly employed for older children in some countries (India, Vietnam, Kenya, Brazil, Nigeria and South Africa). School-based hearing screening focuses only on children suspected of hearing loss (ie, poor academic performance and poor attention skills).^62^ Such targeted screening of children with poor academic performance was reported as a resource-saving method. Additionally, in some countries like South Africa and Kenya, mHealth-based screening was used effectively to screen infants as young as 2 years to older children up to 17 years. They engaged community health workers and trained volunteers as screening personnel, and whenever professional resources were limited, they advocated that these volunteers undertake diagnostic tests under supervision.^37 39 44 59 75^ These are some examples of finding alternative solutions specific to the region’s resources to achieve better outcomes.

### Hearing screening methods

The majority of countries lacked a standardised process or sought to adopt the JCIH protocol.^80 84^ Only a few countries, including South Africa, Brazil, Jordan and Iran, adapted the JCIH protocol to develop a national-level hearing screening policy statement.^85 88^ EHDI, on the other hand, is not legislated as a national policy in any of these countries.^84 89^ A similar trend was observed in Asian LMICs,^19^ where most countries attempted to follow JCIH protocol but were not routinely available as a mandate. The lack of uniform methods across nations further limits the availability of data that can be used to assess EHDI results at the country level.^9 15^

Overall, there was a tendency to adhere to JCIH criteria for EHDI as several studies reported this as the reference benchmark that they adapted^24 51 54 78^ yet reported difficulty in adhering to it.^35 78^ While attempting to meet a benchmark (1, 3, 6 previously and now 1, 2, 3) designed by and for HICs is a good aspiration, such adherence nevertheless necessitates comparable resources and contexts. As a result, there is a simultaneous need to investigate context-specific techniques with impact assessments that can result in cost-effective methods suitable to LMICs regions. Targeted screenings are considered an alternative to universal screening when resources are limited^27 55^; however, the current study found that programme planners mostly attempted universal screening, with relatively few employing targeted screening. This decision is highly determined by the available resources, which in turn influences the overall efficiency, number of babies screened, cost outcomes and follow-up.^85^

Older child screening relied largely on subjective assessments, like in other Asian LMICs.^19^ They were not mandated; hence, there was no common protocol.^86^ While objective screening is preferred, short-term initiatives based on well-conducted pilot studies that use questionnaires, behavioural techniques and/or physiological markers could be adopted.^7^ A few studies, for example, employed questionnaires as screening techniques to identify children suspected of having hearing loss and only performed further screening tests on those identified through the questionnaire.^60 65 74^

Audiologists were most frequently involved in hearing screening of neonates and infants in these countries,^90^ like their Asian counterparts, while nurses routinely performed screening in HICs. Such a trend in LMICs must be investigated further to understand the rationale for using professionals who are scarce in these regions to do a basic screening and the implications for sustainability.

On the other hand, screening for older children was done by community health workers at schools or camps. This differs from the practices observed in Asian LMICs and HICs, where audiologists, school teachers or nurses conduct school screening.^90^ Task shifting to community health workers has been advocated as an appropriate technique in low-resource settings with the scarcity of hearing healthcare experts.^39 59^ Some investigations have shown that task shifting can also be used to perform automated diagnostic pure tone audiometry on older children, where otolaryngologists monitor and analyse the results.^31 66 67^

### Diagnostic methods

A few studies on neonates and babies found significantly high referral rates. The reasons were attributed to various reasons such as the short screening time after birth (<6 hours), noisy screening site and unsuitable equipment.^33 34^ This is significantly higher than the criteria set by JCIH (<4%). Similarly, in older children, noisy screening site, reliance on behavioural response and prevalent ear pathologies like wax impact and OME^43 50 59^ were the causes for higher refer rates. While HICs have achieved satisfied benchmarks, LMICs including the Asian study still report similar high referral rates as a contributing limitation.^91 92^

While programmes that successfully track the progress of identified children have been documented in HICs and Asian LMICs, they are scarce in non-Asian LMICs. One significant challenge noted in several studies was a failure to follow-up for second screening and diagnostic testing.^16 24 34 47 60 63 86 87^ The reasons were a lack of financial support to obtain these services, transportation to the testing site and parental understanding of the importance of early detection and rehabilitation. Another factor was the excessive wait times for diagnostic testing sessions and insufficient follow-up strategies. Some studies attribute the loss of follow-up to the high rate of infant mortality in their regions. The lack of effective follow-up and monitoring systems to complete all the stages of the programme seems to be a significant challenge.^85^

The goal of an EHDI programme is to lower the age of identification so that intervention can begin within the critical period.^85^ Some analyses of EHDI programmes in HICs suggest that the age of identification is around 5 weeks.^18 80^ However, the benefit of a hearing screening programme in lowering the age of identification in LMICs is unknown.^16 19 87^

Diagnostic ABR alone was used to estimate thresholds in neonates/infants, while subjective assessment with otoscopy was performed in older children as young as 2 years old. While WHO (2021) and JCIH (2019) recommend a test battery that includes ABR/ASSR, tympanometry, auditory reflex testing, otoscopy and a medical examination, this has not always been feasible in LMICs.^93^ This needs additional considerations as it is beyond the affordability of the screening programme.

While audiologists performed diagnostic testing on neonates/infants, capacity limitations were overcome in these countries by training community health workers and nurses to perform subjective tests such as pure tone audiometry to diagnose older children.

The prevalence was estimated regionwise as per the World Bank classification and was similar to prevalence rates reported by WHO.^10^ These data, however, should be viewed with caution owing to limited studies, lack of information on prevalence based on hearing thresholds, and small sample size. Another major limitation is the lack of data on the type and degree of hearing loss. The variations in hearing loss classifications also make it difficult to associate the criteria with standards given in HICs. So, the prevalence rates also varied considerably across studies, as reported previously as well.^9^

### Intervention methods

In HICs, follow-up for intervention was usually within 3 months of identification, and the maximum age of intervention was 13.5 months.^80^ However, such information could not be gathered from LMICs as aspects of the intervention were reported in very few studies.^26 27 40 41 43 58 61 66 67 69 71 73^ Information on children who availed of interventions, including HAs, CIs and aural rehabilitation, as well as the age of intervention, was not available in the studies. The outcome of a hearing screening programme is complete only when the child receives appropriate rehabilitation or treatment.^13^ Due to the lack of accessible resources to support children and their families with hearing loss, the number of children receiving these interventions is lower.^14 16 55^ Hence, it is unclear if the expected outcomes/goals of EHDI are met in these regions. This is similar to many other studies that quote the lack of treatment-related information as one of the major limitations of the screening programmes.^16 80 86 87^

### Strengths and limitations

Overall, this systematic review is the first known effort to understand the outcomes of hearing screening programmes in LMICs, including sub-Saharan Africa, the Middle East, Latin America and the Caribbean. This study adheres to all the required guidelines for a systematic review (PRISMA, CASP and RoB). However, there are some limitations to consider, such as the fact that we did not eliminate any articles based on the RoB assessment and that, due to data heterogeneity, we could only perform a narrative synthesis rather than a meta-analysis. Another limitation is that we did not restrict the review to include only studies that identify permanent hearing loss. Hence, the results should be viewed with caution as it includes all types and degrees of hearing loss. Furthermore, publication bias is probable as not all hearing screening projects in LMICs may have published their data in English, given the diversity of native languages in these countries.

## Conclusion

According to the findings of this systematic review, there are attempts towards EHDI in non-Asian LMICs. Overall, studies focused on the screening components of the programme, whereas the diagnostic and intervention aspects were not sufficiently explored. Within countries, the screening procedure, screening instruments, screening personnel, diagnostic personnel, diagnostic tests and testing sites were not standard. Although the protocols used were mostly comparable to those of HICs, only a few countries established their own. However, long-term outcomes in terms of rate of identification, enrolment in appropriate intervention and outcomes are still unknown.

EHDI programme’s long-term viability is dependent on the successful execution of applicable protocols that are appropriate for the local context. It is important to have national procedures (standard protocols and uniform reporting) consistent across the country and linked to current healthcare, social and educational systems. To effectively campaign for such policy changes, studies on hearing screening programmes in LMICs must demonstrate clear outcomes of early rehabilitation.

## supplementary material

10.1136/bmjpo-2024-002794 online supplemental figure 1

10.1136/bmjpo-2024-002794 online supplemental table 1

## Data Availability

Data sharing not applicable as no datasets generated and/or analysed for this study.
