# Supplementary material for: Early Hearing Detection and Intervention programmes for neonates, infants and children in non-Asian low-income and middle-income countries: a systematic review
Source: BMJ Paediatr Open. 2024 Nov 5;8(1):e002794. doi: 10.1136/bmjpo-2024-002794 (PMC11552602; doi:10.1136/bmjpo-2024-002794)
Supplement: online supplemental figure 1 [file bmjpo-8-1-s001.pdf]

**SUPPLEMENTARY MATERIAL FIGURE 1**

a. Results of RoB assessment of the studies describing the neonatal hearing screening programs

| | Q1 – Was there a clear question for the study to address? | Q5 – Is the disease status of the tested population clearly described? | Q6 – Were the methods for performing the test described in sufficient detail? | Q9 – Can the results be applied to your patients/the population of interest? | Q10 – Can the test be applied to your patient or population of interest? | Q11 – Were all outcomes important to the individual or population considered? | Overall |
|---|---|---|---|---|---|---|---|
| A1 | Low | Low | Low | Low | Low | Low | Low |
| A2 | Low | Low | Low | Low | Low | Low | Low |
| A3 | Low | Low | Unclear | Low | Low | Low | Low |
| A4 | Low | Low | Low | Low | Low | Low | Low |
| A5 | Low | Low | Low | Low | Low | Low | Low |
| A6 | Low | Low | Low | Low | Low | Low | Low |
| A7 | Low | Low | Unclear | Low | Low | Low | Low |
| A8 | Low | Low | Low | Low | Low | Low | Low |
| A9 | Low | Unclear | Low | Low | Low | Low | Low |
| A10 | Low | Low | Low | Low | Low | Low | Low |
| A11 | Low | Low | Low | Low | Low | Low | Low |
| A12 | Low | Low | Unclear | Low | Low | Low | Low |
| A13 | Low | Low | Low | Low | Low | Low | Low |
| A14 | Low | Low | Low | Low | Low | Low | Low |
| A15 | Low | Low | Low | Low | Low | Low | Low |
| A16 | Low | Low | Low | Low | Low | Low | Low |
| A17 | Low | Low | Low | Low | Low | Low | Low |
| A18 | Low | Unclear | Low | Low | Low | Low | Low |
| A19 | Low | Unclear | Low | Low | Low | Low | Low |
| A20 | Low | Unclear | Unclear | Low | Low | Low | Low |
| A21 | Low | Low | Low | Low | Low | Low | Low |
| A22 | Low | Low | Low | Low | Low | Low | Low |
| A23 | Low | Low | Low | Low | Low | Low | Low |
| A24 | Low | Low | Low | Low | Low | Low | Low |
| A25 | Low | Low | Low | Low | Low | Low | Low |
| A26 | Low | Low | Low | Low | Low | Low | Low |
| A27 | Low | Unclear | Low | Low | Low | Low | Low |
| A28 | Low | Low | Low | Low | Low | Low | Low |
| A29 | Low | Unclear | Low | Low | Low | Low | Low |

b. Results of RoB assessment of the studies describing the hearing screening programs for infants and older children

| | Q1 – Was there a clear question for the study to address? | Q5 – Is the disease status of the tested population clearly described? | Q6 – Were the methods for performing the test described in sufficient detail? | Q9 – Can the results be applied to your patients/the population of interest? | Q10 – Can the test be applied to your patient or population of interest? | Q11 – Were all outcomes important to the individual or population considered? | Overall |
|---|---|---|---|---|---|---|---|
| B1 | Low | Low | Low | Low | Low | Low | Low |
| B2 | Low | Low | Low | Low | Low | Low | Low |
| B3 | Low | Low | Low | Low | Low | Low | Low |
| B4 | Low | Low | Low | Low | Low | Low | Low |
| B5 | Low | Low | Unclear | Low | Low | Low | Low |
| B6 | Low | Low | Low | Low | Low | Low | Low |
| B7 | Low | Low | Low | Low | Low | Low | Low |
| B8 | Low | Low | Low | Low | Low | Low | Low |
| B9 | Low | Low | Low | Low | Low | Low | Low |
| B10 | Low | Low | Low | Low | Low | Low | Low |
| B11 | Low | Low | Low | Low | Low | Low | Low |
| B12 | Low | Low | Low | Low | Low | Low | Low |
| B13 | Low | Low | Low | Low | Low | Low | Low |
| B14 | Low | Low | Low | Low | Low | Low | Low |
| B15 | Low | Low | Low | Low | Low | Low | Low |
| B16 | Low | Unclear | Low | Low | Low | Low | Low |
| B17 | Low | Low | Low | Low | Low | Low | Low |
| B18 | Low | Unclear | Low | Low | Low | Low | Low |
| B19 | Low | Low | Low | Low | Low | Low | Low |
| B20 | Low | Low | Low | Low | Low | Low | Low |
| B21 | Low | Low | Low | Low | Low | Low | Low |
| B22 | Low | Low | Unclear | Low | Low | Low | Low |
| B23 | Low | Low | Low | Low | Low | Low | Low |
| B24 | Low | Low | Low | Low | Low | Low | Low |
| B25 | Low | Low | Low | Low | Low | Low | Low |
| B26 | Low | Low | Unclear | Low | Low | Low | Low |
| B27 | Low | Low | Unclear | Low | Low | Low | Low |
