# Supplementary material for: Early Hearing Detection and Intervention programmes for neonates, infants and children in non-Asian low-income and middle-income countries: a systematic review
Source: BMJ Paediatr Open. 2024 Nov 5;8(1):e002794. doi: 10.1136/bmjpo-2024-002794 (PMC11552602; doi:10.1136/bmjpo-2024-002794)
Supplement: online supplemental table 1 [file bmjpo-8-1-s002.pdf]

SUPPLEMENTARY MATERIAL TABLE 1

Search strategy and list of databases searched for this review

Database	Key terms used: Participants	Intervention/Exposure	Outcomes	Language	Year	Further Restrictions
PubMed, Web of science, Embase, Scopus, EBSCOHost (Health Business Elite), Google scholar, EBSCO-CINAHL.	Children OR Child OR Newborns OR Infants OR Neonates OR Pre-schoolers OR Pediatrics OR Young children AND developing countries OR Low Middle-Income Countries OR Low-income countries OR Middle-income countries OR Angola OR Argentina OR Burundi OR Burkina Faso OR Belize OR Bolivia OR Brazil OR Botswana OR Central African Republic OR Cote d'Ivoire OR Cameroon OR Congo Democratic republic OR Congo Republic OR Colombia OR Comoros OR Cabo Verde OR Costa Rica OR Cuba OR Djibouti OR Dominica OR Dominican Republic OR Algeria OR Ecuador OR Egypt OR Eritrea OR Ethiopia OR Gabon OR Ghana OR Guinea OR Gambia OR Guinea-Bissau OR Equatorial Guinea OR Grenada OR Guatemala OR Honduras OR Haiti OR Iran OR Iraq OR Jamaica OR Jordan OR Kenya OR Lebanon OR Liberia OR	Neonatal screening OR Hearing tests OR otoacoustic emissions OR Automated auditory brainstem response OR neonatal hearing screening OR newborn hearing screening OR infant hearing screening OR Pre-school hearing screening OR disability screening OR EHDI OR Early Hearing Detection and identification OR Community based screening OR Tele-based hearing screening OR School entry level hearing screening OR Mass screening OR early diagnosis OR early intervention OR Rehabilitation	Congenital hearing loss OR Hearing loss OR hearing impairment OR deafness OR Hearing disorders OR Hearing health OR Deaf	English	2010-2023	

	Libya OR St. Lucia OR Lesotho OR Morocco OR Madagascar OR Mexico OR Mali OR Mozambique OR Mauritania OR Mauritius OR Malawi OR Namibia OR Niger OR Nigeria OR Nicaragua OR Peru OR Paraguay OR West Bank and Gaza OR Rwanda OR Sudan OR Senegal OR Sierra Leone OR El Salvador OR Somalia OR South Sudan OR Sao Tome and Principe OR Suriname OR Eswatini OR Syrian Arab Republic OR Chad OR Togo OR Tunisia OR Tanzania OR Uganda OR St. Vincent and the Grenadines OR Yemen Rep. OR South Africa OR Zambia OR Zimbabwe					
