# Supplementary material for: Early Hearing Detection and Intervention programmes for neonates, infants and children in non-Asian low-income and middle-income countries: a systematic review
Source: BMJ Paediatr Open. 2024 Nov 5;8(1):e002794. doi: 10.1136/bmjpo-2024-002794 (PMC11552602; doi:10.1136/bmjpo-2024-002794)
Supplement: Supplementary file 3 [file bmjpo-2024-002794.reviewer_comments.pdf]

PEER REVIEW HISTORY

BMJ Paediatrics Open publishes all reviews undertaken for accepted manuscripts. Reviewers are asked to complete a checklist review form and are provided with free text boxes to elaborate on their assessment. These free text comments are reproduced below.

ARTICLE DETAILS

**TITLE (PROVISIONAL)**	Early Hearing Detection and Intervention programs for neonates, infants and children in Non-Asian Low- and Middle-Income Countries – A Systematic Review
**AUTHORS**	Rajanbabu, Keerthana; Joshi B, Deepashree; Ramkumar, Vidya; Kuper, Hannah; Vaidyanath, Ramya

VERSION 1 - REVIEW

**REVIEWER NAME**	***Hasantha Gunasekera***
**REVIEWER AFFILIATION**	The University of Sydney Faculty of Medicine and Health, CHW Clinical School
**REVIEWER CONFLICT OF INTEREST**	
**DATE REVIEW RETURNED**	28-Jul-2024

**GENERAL COMMENTS**	BMJPaedOpen2024002794<br>Thanks for the opportunity to review this submission. My comments and suggestions are as follows:<br>Abstract is 370 words and unstructured and grey literature search should be mentioned (given it is likely work would be there rather than published in scientific journals for these sorts of public health programs). Not clear why only 2010 onwards selected? Suggest making it clear this is a sister article to the Sys Rev published in this journal in 2023 (otherwise, isn't clear why only non-Asian LMICs!)<br>Intro is not well structure (e.g., 75% preventable vs 46% in HIC in para 1 then 60% figure in para 3. Although you state that after the neonatal period, school is the next opportunity to screen, that misses immunisation opportunistic screening (as mentioned in discussion)<br>L18: China is not in the LMIC category any more is it?<br>Methods: you mention narrative review but this is described in the rest (incl Title and Discussion) as a systematic review. I think it is important to go into more detail on the difference between neonatal (mostly SNHL) vs. infant/adolescent (mostly conductive) and unilateral (not as clear this causes functional impairment) vs. bilateral (ie functional impairment) and degree of severity (not very different cut-off categories used with various definitions).<br>Results:<br>1. Suggest adding number of countries included in review and denominator in results and abstract rather than discussion.<br>2. For neonatal tympanometry screening, was this a 1kHz probe or a standard probe (as standard probes would be unreliable in this age wouldn't they?)<br>3. Universal vs Opportunistic seems like a logical heading under which to report<br>4. Fig 2 Hard to read- do you need to show the negative prevalence side of this graph? The individual Forest Plots could be labelled with countries rather than as footnotes to make it easier to read. The

	footnotes should be the type of “hearing loss” described as that can be very different, conductive vs. sensorineural, the various different definitions (incl recent WHO World Hearing Report vs prev definitions) of severity and whether bilateral (Hearing impairment) or unilateral (ie hearing loss measured in one ear but the other ear normal, which is potentially very different functionally).<br>5. Supplementary Fig 1: I am sceptical that all the studies have mainly low ROB for all parameters!<br>6. Supplementary Table 2 is large but actually very important data so suggest include in main article<br>Typos/minor comments<br>1. Pg 6 L9: Age of 15 years<br>2. L13: not sure only HIC have the knowledge although I agree re resources comment<br>3. Pg12 L3: suggest either report numbers of studies or references but not a mix of both (the easiest would be numbers of countries using the technique reported)

**REVIEWER NAME**	***Elizabeth M. Fitzpatrick***
**REVIEWER AFFILIATION**	None disclosed
**REVIEWER CONFLICT OF INTEREST**	
**DATE REVIEW RETURNED**	29-Jul-2024

**GENERAL COMMENTS**	This paper has the potential to act as a good complimentary contribution to a previously published systematic review published in 2023 related to EHDI programmes in LMIC countries in Asia. The authors have followed appropriate guidelines (e.g., PRISMA) for conducting systematic reviews and overall, the review appears to be methodologically sound.<br>However, the present reporting structure makes the overall findings somewhat difficult to follow. Specific recommendations and suggestions are provided.<br><br>Abstract.<br>-Lines 9-13. It would be helpful to give the reader some idea of when EHDI were established. E.g., last three decades…<br>-Something a little more specific in terms of inclusion criteria would be helpful.<br>-Line 43 – Results. If space permits, please briefly summarize type of studies, e.g. cohort studies, etc.<br>-Line 45. State n for newborn hearing screening (NHS) and for school-based programs.<br>-Line 54. Audiologists for both NHS and older children?<br><br>Introduction.<br>Page 6. -Line 51. How good is the evidence here? Please state something about the findings of these studies.<br>Page 7.<br>-Line 14. Not just acquired - also later onset hearing loss (HL)<br>-Line 15. Awkward phrasing – please rephrase. E.g.,, When newborn hearing screening is not universally implemented, or children are missed during the screen …<br>-Line 52. The term follow-up rate is used throughout, but I wonder if the authors are referring to refer rate – this would be an a very important metric to capture, i.e., % of children referred to diagnostic audiology from the screening program. Collecting follow-up rate (how many actually followed up with their diagnostic audiometry

	assessment after referral) would be useful too but this is typically more difficult to document. Please clarify.<br>Same comment applies to intervention data on top of next page.<br>Page 8.<br>The inclusion criteria states children < age 6. However, in this review, data are also extracted on school-age screening – was it limited to school-age children < age 6 years?<br>-Please provide justification for the start year – 2010 – was this arbitrary?<br>-Line 43. Please rephrase – the study inclusion dates were 2010-2013 but presumably, that is not when the search was conducted.<br>-Was the search limited to screening only or were programs implementing early detection and intervention also included (independent of whether NHS was in place?).<br>Page 9.<br>-Line 9. Please clarify – title screening – i.e., was it the first step and conducted by 2 reviewers prior to screening title and abstracts. Please clarify the steps.<br>-Line 36. Specify what is meant by identification rate and intervention rate. Is this the refer rate from the screen to diagnostic assessment and then for intervention after audiologic assessment?<br>Page 10.<br>Were the scoring system decisions re bias for the individual CASP questions made by the authors (i.e., authors' own ratings or are they defined in the CASP checklist? Same question for the overall rating of bias.<br>Page 10 and onwards: - Results: The primary concern I have is the organization of the results section – it is difficult to follow given that NHS and post-neonatal /school screening are presented together. These are typically two very different kinds of screening programs. The authors start out by presenting NHS and older children in two separate Tables. I would recommend that the findings be presented in this manner (i.e., the 29 studies related to NHS separately, 26 related to infant/older children) in the text to make it easier for the reader to follow. Consider whether it would be preferable to present NHS and infant (what constitutes infant in your categorization?) together and then 'older' children.<br>-Lines 43-49. Please categorize the countries by low and middle-income.<br>Page 11.<br>-Lines 3-4. Please explain which items (4 of how many) were eliminated and why.<br>-Line 50. We see here that studies with children up to age 17 years were included. Revise the description of inclusion criteria. Perhaps organize criteria according to NHS and post-natal + school-age screening.<br>Page 12.<br>-Paragraph starting at Line 38. Presumably, test methods apply to older children, but it is not specifically stated. Again, re-structuring the text according to NHS and other would be more useful.<br>-Line 53. Is this section referring to school-age only?<br>Page 14.<br>-Line 37-38. Only 16 of the 29 screening programs had diagnostic services taking place in a hospital setting - is that correct? Could the authors please make that more explicit.<br>Page 15.<br>-Line 54. This prevalence seems very high. Could this number be because transient conductive HL was also included in some studies? Did the studies define PERMANENT hearing loss?<br>Page 16.

	- Line 18. Same question applies re inclusion criteria.<br>-Please also be very clear in the inclusion criteria at the beginning as to whether you are searching for articles on permanent hearing loss only. Please define permanent hearing loss.<br>-Line 30. This implies that conductive HL was part of the target disorder. Again, please clarify in inclusion criteria. Because the authors refer to NHS in HICs and JCIH guidelines, it implies that the target disorder is permanent HL.<br>-Line 42. Same question as above re follow-up rate.<br>Page 17.<br>-Line 18. Not clear - the screening program provided these medical services??<br>-Line 52. Did the authors of the studies report these as cost-effective measures or is this the conclusion of the authors of this review? i.e., were these 5 studies cited here specifically examining the use of different personnel from a cost perspective? Please clarify.<br>-Page 18.<br>-Line 38-42. Did the authors examine whether or which WHO criteria were applied in the included studies?<br>-Line 50. State how many so that the reader does not have to check back in the results section.<br>-Lines 52-53. Please clarify the statement.<br>-Paragraph starting on line 48. The paragraph a little difficult to follow - as often only one study is cited to support a point; Perhaps the authors could start by making a point about alternative solutions, and how NHS is adapted - and then report these as examples of adaptations.<br>-Page 20, Line 38. The authors refer frequently to JCIH criteria. However, I do not see any discussion of JCIH benchmarks (1-3-6), could a brief statement be added to inform the reader as to whether these were reported in any of the included studies?<br>-Line 52. I'm not sure that targeted screening programs are clearly differentiated from universal screening initiatives in the Results section.<br>Page 21.<br>-Lines 18-24. Please comment on diagnostic referral rate. Is it reported in any studies; is it comparable to that reported for HICs?<br>-Line 30. This is unclear - was otoscopy only being used to carry out a hearing assessment? this needs to be clear in results - how many studies?<br>Page 22. -Line 17. Requires citations of larger NHS studies.<br>Page 23. Line 16. I don't believe this information was reported in the findings – i.e., info on NHS led by private or NGO-type organizations versus those led by government initiatives.<br><br>Several minor edits are required (a few examples are provided below).<br>Line 15. Delete 'the' before EHDI programs.<br>Line 26. Revise: e.g. Is there a word missing? These programs largely follow the protocols or practices? of HICs….<br>Line 40. Delete 'the' before stakeholders and before LMICs<br><br>Abstract<br>Line 9. Add years after 15.<br><br>Introduction<br>Line 9 – 15 years.<br>Line 26. Delete 'some'<br><br>Page 9. Line 38. Data were…

	Etc.

VERSION 1 – AUTHOR RESPONSE

Reviewer 1

	**Comment**	**Reason**	**Modifications made in manuscript**
Abstract	It appears that the provided abstract in the system and in the manuscript is not similar. Please be informed that this information should match in both main document and in the submission screen. Kindly ensure that the contents in the both fields should be identical.	Due to the word limit of 300, the abstract was cut short to 300 words during the manuscript submission.	Yes, this has been corrected in the manuscript. Page 1-2
	370 words and unstructured and grey literature search should be mentioned (given it is likely work would be there rather than published in scientific journals for these public health programs)	-	This has been added.<br><br>Page 1, Para 2
	Not clear why only 2010 onwards selected?	Since the literature is more than a decade old and is often not relevant, we restricted the search to 2010.	-
	Suggest making it clear this is a sister article to the Sys Rev published in this journal in 2023 (otherwise, isn't clear why only non-Asian LMICs!)	It was mentioned only in the Intro and not the abstract due to the word limit restriction.	This has been mentioned.<br><br>Intro – Page 4, Last para
Intro	not well structured (e.g., 75% preventable vs 46% in HIC in para 1 then 60% figure in para 3	-	This has been modified.<br><br>Page 3 "Among the preventable causes, nearly 60% of them is attributed to poor maternal nutrition and hygiene and late detection

			and treatment of otitis media [2]”
	Although you state that after the neonatal period, school is the next opportunity to screen, that misses immunization opportunistic screening (as mentioned in discussion)	-	This has been added.<br><br>Introduction Page 4, Para 2
	L18: China is not in the L MIC category anymore, is it?	The expansion for LMIC is Low- and Middle-Income Country. China is a middle-income country.<br><br>Hence, mentioned.	-
Methods	you mention narrative review but this is described in the rest (incl Title and Discussion) as a systematic review	It is a systematic review.<br><br>A narrative synthesis was done to report the extracted data, and forest plots were generated to estimate the pooled prevalence.	This has been clarified.<br><br>Abstract Page 1 Para 2<br><br>Method (Data extraction and synthesis) Page 6-7
	I think it is important to go into more detail on the difference between neonatal (mostly SNHL) vs. infant/adolescent (mostly conductive) and unilateral (not as clear this causes functional impairment) vs. bilateral (ie functional impairment) and degree of severity (not very different cut-off categories used with various definitions)	While attempts were made to collect both type and degree of hearing loss, only a very few studies have these data.<br><br>Hence, it is mentioned in the limitation.	Discussion Page 30
Results	1. Suggest adding number of countries included in review and denominator in results and abstract rather than discussion	-	It has been added.<br><br>Abstract (Page 1, Para 3) “Fifty-six studies from 16 LMICs...”

			Results (Page 16, Para 1) "Studies were obtained from 16 LMICs of the non-Asian regions".
	2. For neonatal tympanometry screening, was this a 1kHz probe or a standard probe (as standard probes would be unreliable in this age, wouldn't they?)	2 studies mentioned using a 1KHz tone as they are more sensitive for neonates.<br><br>There was no mention of probe tone in one study (Wong et al).	-
	3. Universal vs Opportunistic seems like a logical heading under which to report	We chose to classify the screening programs based on age, as, in the LMIC context, none of the programs are universal.	-
	4. Fig 2 Hard to read- do you need to show the negative prevalence side of this graph? The individual Forest Plots could be labelled with countries rather than as footnotes to make it easier to read. The footnotes should be the type of "hearing loss" described as that can be very different, conductive vs. sensorineural, the various different definitions (incl recent WHO World Hearing Report vs previous definitions) of severity and whether bilateral (Hearing impairment) or unilateral (ie hearing loss measured in one ear but the other ear normal, which is potentially very different functionally)	Many studies did not mention the type and degree of hearing loss.	Figure 2 (Forest plot of prevalence rates) has been labeled within the figure.<br><br>Results Page 21-22
	5. Supplementary Fig 1: I am skeptical that all the studies have mainly low ROB for all parameters!	The RoB assessment was done using the CASP questionnaire, which has a standard	-

		set of questions related to the structure of the study included.<br><br>Most of the studies answered the questions included in the questionnaire. They have directly addressed any limitations, as asked in the CASP questions. The ROB is low since the scoring only asks if the information is present.	
	6. Supplementary Table 2 is large but actually very important data so suggest include in main article	Yes, it was not added in the main document earlier due to word limits. However,<br><br>We agree with the reviewer's comment on the table and have included it in the main document.	Tables 1a & 1b are included in the results (Pages 8-15)
Typos/minor comments	Pg 6 L9: Age of 15 years	-	It has been added.
	L13: not sure only HIC have the knowledge although I agree re resources comment	Not understandable	-
	Pg12 L3: suggest either report numbers of studies or references but not a mix of both (the easiest would be numbers of countries using the technique reported)	The citations/references were included since it would be easy for the reader to refer back to a particular included study if required.	-

Reviewer 2

	**Comment**	**Reason**	**Modifications made in manuscript**
Abstract	-Lines 9-13. It would be helpful to give the reader some idea of when EHDI	Thank you for the suggestion. However, considering the word	-

	were established. E.g., last three decades…	limit, the introduction has this information. Since it's an abstract, we have chosen to exclude this information.	
	Something a little more specific in terms of inclusion criteria would be helpful.	Thank you for the suggestion. However, due to the word limit, it couldn't be added to the abstract.	-
	Line 43 – Results. If space permits, please briefly summarize type of studies, e.g. cohort studies, etc	Thank you for the suggestion. However, due to the word limit, it couldn't be added to the abstract.	-
	Line 45. State n for newborn hearing screening (NHS) and for school-based programs	-	This has been added.<br><br>Abstract (Page 1, Para 3) – "… grouped into 29 hearing screening programs for neonates and infants and 26 hearing screening programs for older children."
	Line 54. Audiologists for both NHS and older children?	For the neonatal screening group. Community health workers were employed to screen the older children.	It has been modified.<br><br>Page 1, Para 3 – "… audiologists performed screening and diagnostics for neonates while community health workers were the screening personnel for older children."
Introduction	Page 6. -Line 51. How good is the evidence here? Please state something about the findings of these studies	Citation 7 is a WHO report<br><br>based on several countries, and citation 10 is a commentary highlighting the WHO report on NHS.<br><br>So, they suggested alternatives or solutions to implement. The	-

		difficulties are mentioned in the next few lines, and the solutions are discussed.	
	Page 7. -Line 14. Not just acquired - also later onset hearing loss (HL)	-	It has been added.<br><br>Page 4
	Line 15. Awkward phrasing – please rephrase. E.g.,, When newborn hearing screening is not universally implemented, or children are missed during the screen …	-	It has been modified.<br><br>Page 4, Para 2 – "When children miss the initial screenings at the place of birth, like hospitals and public health centers, or when screening at birth is not implemented universally in their region, school screenings are the next possible level of early identification."
	Line 52. The term follow-up rate is used throughout, but I wonder if the authors are referring to refer rate – this would be an a very important metric to capture, i.e., % of children referred to diagnostic audiology from the screening program. Collecting follow-up rate (how many actually followed up with their diagnostic audiometry assessment after referral) would be useful too but this is typically more difficult to document. Please clarify. Same comment applies to intervention data on top of next page.	Follow-up rate here refers to the number of people following up for diagnostic assessment.<br><br>The intervention rate refers to the number of individuals reported to have undergone some kind of intervention as a part of the program. This information was extracted if reported.<br><br>Yes, we agree. We have added the refer rate details to the extraction table and mentioned them in the Results as well.	Table 1a & 1b (Page 8-15)<br><br>Results (Page 19-20)
	Page 8 - The inclusion criteria states children < age 6. However, in this review, data are also extracted on school-age screening – was it limited to	-	Inclusion criteria have been modified.<br><br>Methodology Page 5, Para 2

	school-age children < age 6 years?		
	Please provide justification for the start year – 2010 – was this arbitrary?	Since the literature is more than a decade old and often irrelevant, we restricted the search to 2010.	-
	Line 43. Please rephrase – the study inclusion dates were 2010-2013 but presumably, that is not when the search was conducted	-	It has been modified<br><br>Page 5, Last para "The search was conducted from late 2022 till mid-2023 for studies between January 2010 to March 2023."
	Was the search limited to screening only or were programs implementing early detection and intervention also included (independent of whether NHS was in place?)	The search included all studies that had hearing screenings. The MeSH terms included hearing screening, EHDI, and NHS.	-
	Page 9 - Line 9. Please clarify – title screening – i.e., was it the first step and conducted by 2 reviewers prior to screening title and abstracts. Please clarify the steps.	Yes, title screening was the first step and was done by only two reviewers. The abstract screening was the second step done by 2 reviewers	It has been modified.<br><br>Page 6
	Line 36. Specify what is meant by identification rate and intervention rate. Is this the refer rate from the screen to diagnostic assessment and then for intervention after audiologic assessment?	The definition of refer rate, the identification rate, and the intervention rate have been mentioned.	This has been included.<br><br>Data extraction and Synthesis<br><br>Page 6 – " … refer rate (number of children referred from screening stage to diagnostic testing) … identification rate (number of children identified to have hearing loss out of the total screened population), intervention rate (number of children who received intervention out of the children identified with hearing loss)"

	Page 10 - Were the scoring system decisions re bias for the individual CASP questions made by the authors (i.e., authors' own ratings or are they defined in the CASP checklist? Same question for the overall rating of bias.	The scoring for CASP questions was as per the CASP rating guidelines. The overall bias criteria were set by the authors.	Page 7 – "This criterion for labeling overall bias as high/medium/low was arbitrarily developed by the authors."
Results	Page 10 and onwards:<br><br>The primary concern I have is the organization of the results section – it is difficult to follow given that NHS and post-neonatal /school screening are presented together. These are typically two very different kinds of screening programs. The authors start out by presenting NHS and older children in two separate Tables. I would recommend that the findings be presented in this manner (i.e., the 29 studies related to NHS separately, 26 related to infant/older children) in the text to make it easier for the reader to follow.	Under each heading in the results, Hearing screening for neonates and infants is described in 1st half; hearing screening for older children is described in 2nd half.<br><br>Since the tables were previously given as supplementary material, the readability of the text was difficult.<br><br>Now,<br><br>We hope the inclusion of the tables within the manuscript clarifies the concern.	It has been modified.<br><br>The tables for hearing screening for neonates and infants (Table 1a) and hearing screening for older children (Table 1b) are included in the results Page 8-15.
	Consider whether it would be preferable to present NHS and infant (what constitutes infant in your categorization?) together and then 'older' children.	Yes, we agree with the reviewer. The content was already represented in this manner. However, there was an error in labeling the context. Now it has been corrected	Modifications were made to the title according to the groups.<br><br>The screening program titles based on the age range are as follows<br><br>i)below 1 year: "Hearing Screening for neonates and infants"<br><br>ii) above 1 year: "Hearing Screening for older children".

	Lines 43-49. Please categorize the countries by low and middle-income	Yes, they have been categorized based on region and number of studies now.	It has been added.<br><br>Page 16, Para 1
	Page 11. -Lines 3-4. Please explain which items (4 of how many) were eliminated and why	-	This has been added.<br><br>Page 16 – "…four (out of 12). … studies do not have any reference standards or cost alternatives. So, these questions were deemed not applicable."
	Line 50. We see here that studies with children up to age 17 years were included. Revise the description of inclusion criteria. Perhaps organize criteria according to NHS and post-natal + school-age screening.	-	The inclusion criteria have been modified.<br><br>Page 5
	Page 12. -Paragraph starting at Line 38. Presumably, test methods apply to older children, but it is not specifically stated.<br><br>Again, re-structuring the text according to NHS and other would be more useful.	Thanks for the suggestion	It has been modified.<br><br>Results Page 18<br><br>"Otoscopy and pure tone audiometry were the most used tests for older children".
	Line 53. Is this section referring to school-age only?	Yes (continuation of the previous paragraph)	It has been modified.<br><br>Results Page 18<br><br>"In addition, Validated questionnaires [40,60,61,66], SIFTER [74], and LittleEars questionnaire [65]) were also used for screening for older children."
	Page 14. -Line 37-38. Only 16 of the 29 screening programs had diagnostic services taking place in a hospital setting - is that correct? Could the authors	Yes. This is correct. This has been made explicit in the text	It has been modified.<br><br>Results Page 20<br><br>"Only in 16 of the 29 studies for neonates and infants, diagnostic testing

	please make that more explicit.		was conducted in a tertiary care hospital (n=16), which was different from the screening site”
	Page 15-Line 54. This prevalence seems very high. Could this number be because transient conductive HL was also included in some studies? Did the studies define PERMANENT hearing loss?	Yes, this is possible. the review included all studies irrespective of the type and degrees they mentioned.<br><br>Because there was no mention/proper definition of permanent hearing loss in the studies included.<br><br>This was also mentioned in discussion as a limitation.	Modified in the text.<br><br>Results Page 21-22<br><br>Discussion Page 30
	Page 16 - Line 18. Same question applies re inclusion criteria.<br>-Please also be very clear in the inclusion criteria at the beginning as to whether you are searching for articles on permanent hearing loss only. Please define permanent hearing loss.	The review aims to study all the screening programs that identify hearing loss. There was no restriction on the type of hearing loss the study screened. Data on the type of hearing loss identified was found only after the results and analysis.	It has been modified.<br><br>Inclusion criteria Page 5<br><br>“The review included programs identifying any type and degree of hearing loss (not restricted to permanent hearing loss).”<br><br>Result (Page 21-22) – More info on type of loss (SNHL, Cond, Uni or Bi)
	Line 30. This implies that conductive HL was part of the target disorder. Again, please clarify in inclusion criteria. Because the authors refer to NHS in HICs and JCIH guidelines, it implies that the target disorder is permanent HL	Yes. Thank you for the suggestion	It has been modified.<br><br>Inclusion criteria Page 5<br><br>“The review included programs identifying any type and degree of hearing loss (not restricted to permanent hearing loss).”
	Line 42. Same question as above re follow-up rate.	Follow-up rate and refer rate have been explained and reported separately.	Definition Page 6<br><br>Results Page 19-20

	Page 17-Line 18. Not clear - the screening program provided these medical services??	Yes, the screening program provided these interventions.	-
	Line 52. Did the authors of the studies report these as cost-effective measures or is this the conclusion of the authors of this review? i.e., were these 5 studies cited here specifically examining the use of different personnel from a cost perspective? Please clarify.	The cost-effectiveness reported is based on the authors of the studies included in this review and not the authors of the review itself.	-
	Page 18-Line 38-42. Did the authors examine whether or which WHO criteria were applied in the included studies?	No, The WHO criteria were not used as a benchmark to map the included studies.	-
	Line 50. State how many so that the reader does not have to check back in the results section.	The school screenings were suggested as the alternative recommendations. The studies in the review did not specifically mention this.	It has been modified.<br>Discussion Page 25<br>"Considering the burden of unidentified hearing loss, school-based screening has been increasingly employed and suggested for older children in many of these countries".
	Lines 52-53. Please clarify the statement.	-	It has been modified.<br>Page 25
	Paragraph starting on line 48. The paragraph a little difficult to follow - as often only one study is cited to support a point; Perhaps the authors could start by making a point about alternative solutions, and how NHS is adapted - and		It has been modified

	then report these as examples of adaptations.		
	Page 20, Line 38. The authors refer frequently to JCIH criteria. However, I do not see any discussion of JCIH benchmarks (1-3-6), could a brief statement be added to inform the reader as to whether these were reported in any of the included studies?	-	It has been added.<br><br>Page 26
	Line 52. I'm not sure that targeted screening programs are clearly differentiated from universal screening initiatives in the Results section.	Yes, Targeted programs are not differentiated since many programs attempted universal screening.	-
	Page 21.<br>-Lines 18-24. Please comment on diagnostic referral rate. Is it reported in any studies; is it comparable to that reported for HICs?	-	It has been added.<br><br>Results Page 20<br><br>Discussion Page 28
	Line 30. This is unclear - was otoscopy only being used to carry out a hearing assessment? this needs to be clear in results - how many studies?	No, subjective assessment and otoscopy together were used in 1st stage to screen. It has been mentioned in the results section.	-
	Page 22. -Line 17. Requires citations of larger NHS studies.	It is a systematic review where age is obtained as a pooled value.	-
	Page 23. Line 16. I don't believe this information was reported in the findings – i.e., info on NHS led by private or NGO-type organizations versus those led by government initiatives.	-	It has been removed.<br><br>Page 30

Several minor edits are required	(a few examples are provided below).<br>Line 15. Delete ‘the’ before EHDI programs.	-	It has been modified.
	Line 26. Revise: e.g. Is there a word missing? These programs largely follow the protocols or practices? of HICs….	-	It has been modified.
	Line 40. Delete ‘the’ before stakeholders and before LMICs	-	It has been modified.
Abstract	Line 9. Add years after 15.	-	It has been added.
Introduction	Line 9 – 15 years.<br>Line 26. Delete ‘some’	-	It has been modified.
	Page 9. Line 38. Data were…		

VERSION 2 – REVIEW

**REVIEWER NAME**	***Hasantha Gunasekera***
**REVIEWER AFFILIATION**	The University of Sydney Faculty of Medicine and Health, CHW Clinical School
**REVIEWER CONFLICT OF INTEREST**	
**DATE REVIEW RETURNED**	24-Sep-2024

**GENERAL COMMENTS**	Thanks for making these amendments. Some terms may need to be changed for the journal (e.g., paediatric vs. pediatric) and "below 6 years" to "younger than 6 years" and "data are (prevalence section) but I am otherwise happy with the responses. I think my comment about HICs in the intro was that they are not the only ones with the knowledge!
